# Oclusão do Apêndice Atrial Esquerdo com a Prótese Lambre: Experiência Multicêntrica Inicial no Brasil

**DOI:** 10.36660/abc.20210275

**Published:** 2022-04-26

**Authors:** Francisco Chamié, Enio Guerios, Daniel Peralta e Silva, Valério Fuks, Rômulo Torres

**Affiliations:** 1 Hospital Federal dos Servidores do Estado Rio de Janeiro RJ Brasil Hospital Federal dos Servidores do Estado – Cardiologia Intervencionista, Rio de Janeiro, RJ – Brasil; 2 INTERCAT Rio de Janeiro RJ Brasil INTERCAT - Cardiologia Intervencionista, Rio de Janeiro, RJ – Brasil; 3 Universidade Federal do Paraná Hospital de Clínicas Curitiba PR Brasil Universidade Federal do Paraná – Hospital de Clínicas, Curitiba, PR – Brasil; 4 Hospital Marcelino Champagnat Curitiba PR Brasil Hospital Marcelino Champagnat, Curitiba, PR – Brasil

**Keywords:** Fibrilação Atrial, Apêndice Atrial, Oclusão Coronária

## Abstract

**Fundamento:**

A oclusão do apêndice atrial esquerdo (AAE) tem se mostrado uma alternativa à terapia de anticoagulação oral (ACO) para prevenção de acidente vascular cerebral (AVC) em pacientes com fibrilação atrial não valvar (FANV).

**Objetivos:**

Descrever os primeiros resultados de uma experiência inicial multicêntrica no Brasil e investigar a viabilidade, a segurança e a eficácia da oclusão do AAE com o novo dispositivo LAmbre.

**Métodos:**

Coletamos dados do procedimento e do acompanhamento de 51 pacientes consecutivos com FANV, restrições para ACO em longo prazo e com anatomia adequada, submetidos à oclusão do AAE com o dispositivo LAmbre em 18 centros no Brasil. Indicações para o procedimento foram: sangramento importante em pacientes recebendo ACO (47,1%), AVC ou trombo persistente no AAE apesar de ACO adequada (27.5%), sangramento e AVC (17.6%), outras contraindicações clínicas apara ACO (5,9%), e escolha do paciente devido à prática esportiva (1,9%).

**Resultados:**

Foram estudados 25 homens (49%) e 26 mulheres (51%), com idade média de 76±7,7 anos, escore CHA2DS2-VASc médio de 4,6± 1,7 e escore HAS-BLED médio de 3.4± 1,1. A taxa de sucesso do procedimento foi de 100%. As complicações imediatas relacionadas ao procedimento foram derrame pericárdico em dois pacientes, e embolização do dispositivo em um caso. Não foram observados shunts residuais > 5mm. Shunts < 5mm foram detectados em quatro pacientes por Doppler colorido ao final do procedimento. Após um período médio de acompanhamento de 18 meses ± 12 meses, não foram observados óbito, AVC ou complicações maiores.

**Conclusão:**

A oclusão do AAE com o dispositivo LAmbre foi segura e eficaz nesta pequena série de casos. Apesar desses resultados iniciais encorajadores, dado o pequeno número de casos, serão necessários mais estudos com um maior período de acompanhamento.

## Introdução

A oclusão transcateter do apêndice atrial esquerdo (AAE) tem se tornado cada vez mais popular como alternativa à anticoagulação para profilaxia de eventos tromboembólicos em pacientes com fibrilação atrial não valvar (FANV).^1^ A terapia de anticoagulação oral (ACO), seja com antagonistas de vitamina K (AVK) ou com anticoagulantes orais diretos (DOAC), é uma terapia efetiva para prevenção de acidente vascular cerebral (AVC) em pacientes com FA.^2^ Infelizmente, a adesão aos DOACs em longo prazo, subprescrição e complicações, impedem a utilização dessa terapia por um grande número de pacientes. Assim, a necessidade de uma forma não farmacológica de profilaxia aumentou no decorrer dos anos.^3^

Considerando que mais de 90% dos trombos atriais formados como consequência da FANV estão localizados na porção trabecular do apêndice atrial, a oclusão do AAE parece ser uma opção razoável.^4^ Inicialmente proposto como um procedimento cirúrgico, a oclusão percutânea é atualmente realizada em todo o mundo, e diferentes dispositivos e técnicas estão disponíveis. Ensaios randomizados mostraram que a oclusão do AAE não é inferior à varfarina e aos DOACs em termos de redução de AVC e embolismo sistêmico, e superior à varfarina quanto à mortalidade tardia.^5 - 10^

O LAmbre é um dispositivo de oclusão do AAE lançado no Brasil em 2018, depois dos dispositivos Amplatzer Cardiac Plug (ACP) e Watchman Filter. O presente artigo tem o objetivo de descrever os resultados do primeiro registro multicêntrico brasileiro do uso do LAmbre para oclusão percutânea do AAE na prevenção de AVC em pacientes com FANV.

## Métodos

Entre maio de 2018 e novembro de 2020, foram estudados, prospectivamente, pacientes consecutivos, submetidos à oclusão de AAE utilizando o dispositivo LAmbre em 18 centros no Brasil. Muitos desses procedimentos foram realizados sob supervisão de um preceptor. Todos os pacientes apresentaram FANV e uma contraindicação relativa ou absoluta para terapia com DOACs em longo prazo – a única exceção foi um paciente que se recusou a receber DOACs por preferência pessoal. Todos os pacientes foram submetidos previamente à ecocardiografia transesofágica (ETE) ou à tomografia computadorizada cardíaca (TCC) para avaliação da dimensão, da morfologia, e da zona de implante do AAE, e presença de trombos.

Os procedimentos foram realizados sob anestesia geral e intubação orotraqueal. Foram administrados heparina não fracionada (100 mg/kg ou 10 000 Unidades Internacionais) e profilaxia com antibiótico (2g de Cefazolina intravenosa) a todos os pacientes, seguido de 1g de Cefazolina intravenosa, dose única, seis horas após o procedimento, na unidade de terapia intensiva (UTI). Os procedimentos foram monitorados por ETE e fluoroscopia.

Depois de se obter acesso venoso femoral, realizou-se punção transeptal com agulha Brockenbrough, posicionando-a na região inferior e posterior da fossa oval. A pressão do átrio esquerdo foi registrada imediatamente após seu acesso; em caso de valores inferiores a 10mmHg, solução salina era infundida rapidamente para restaurar os diâmetros reais do AAE. Um cateter angiográfico *Pigtail* (5F) foi posicionado dentro do AAE para obtenção de medidas em projeção oblíqua anterior direita cranial e caudal. Após angiografia, um fio-guia Super-Stiff J-Tip (0,035”/260 cm) foi cuidadosamente introduzido dentro do AAE através do cateter *Pigtail* . O tamanho do dispositivo foi confirmado por angiografia intraoperatória e ETE e deveria ser 2-8 mm maior que a zona de implantação. O dispositivo foi implantado usando a bainha longa indicada para o tamanho do dispositivo escolhido. A técnica de implante foi descrita previamente.^11^

Os pacientes foram mantidos na UTI durante toda a noite, e tiveram alta hospitalar no dia seguinte após outro ETE, na ausência de complicações.

Foram prescritos aspirina (100 mg) e clopidogrel (75 mg) após o procedimento. O Clopidogrel foi interrompido após três meses, e o uso contínuo da aspirina prescrito em seguida. O ETE de acompanhamento foi realizado aos três meses e seis meses após o procedimento.

### Dispositivo Lambre®

**Figura 1:**
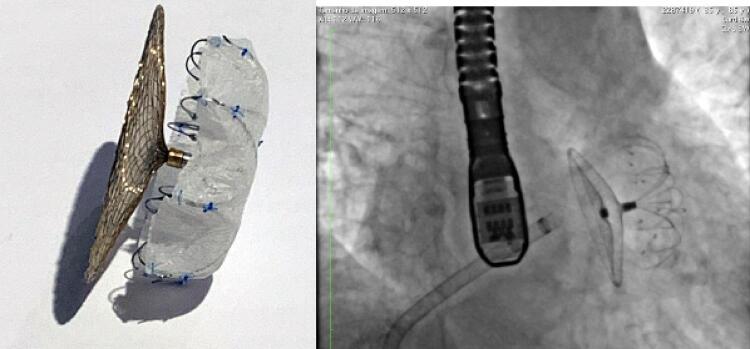
– Dispositivo LAmbre®: guarda-chuva (umbrella) e disco conectados por um pino central (à esquerda). Os braços possuem pontas arredondadas atraumáticas que envolvem a porção trabecular do apêndice atrial esquerdo (AAE) e pequenos ganchos que se fixam à parede do AAE. O disco cobre o óstio do AAE e se conecta ao guarda-chuva por um pino, sem visualização do parafuso na superfície externa do disco. À direita, fluoroscopia após o implante do dispositivo LAmbre®.

O LAmbre® (Lifetech Scientific, Shenzhen, China) é um dispositivo formado por uma malha de nitinol autoexpansível, coberta por nitreto de titânio (TiN). O dispositivo é composto por três partes: um disco que cobre o óstio do AAE, um pino conector, e uma estrutura de fixação composta por 8 braços em formato de guarda-chuva ( *umbrella* ) e pequenos ganchos que prende o dispositivo no corpo do AAE, aumentando a estabilidade. Os braços do umbrella se armam com um movimento de rotação anterógrada. e suas pontas atraumáticas, quando totalmente abertas, envolvem as trabéculas do AAE, e os pequenos ganchos distais se conectam à parede do AAE, aumentando a estabilidade do dispositivo. O disco é configurado para cobrir totalmente o óstio do AAE. Tanto o *umbrella* como o disco possuem tecido de tereftalato de polietileno costurado em seu interior ( Figura 1 ). Ainda, o dispositivo LAmbre® tem duas versões: o modelo padrão e o modelo especial.

No LAmbre® do tipo padrão, o tamanho do *umbrella* varia entre 16 e 36 mm, com aumentos de dois em dois milímetros, com discos 6 mm maiores que os *umbrellas* de 16-30mm ou 4 mm maiores que os *umbrellas* de 32-36 mm. No tipo especial, o tamanho do *umbrella* varia entre 16 e 26 mm, com aumentos de dois em dois milímetros, com discos 14 mm maiores que os *umbrellas* de 16-18 mm ou 12 mm maiores que os *umbrellas* de 20-26 mm.

O sistema de entrega é composto de bainhas com dupla curvatura (45 e 30 graus) com calibres variando de 8F a 10F, e um cabo de entrega com sistema de rosca. Vale ressaltar que o parafuso sobre a superfície do disco fica recuado, para prevenir a formação de trombo sobre o próprio dispositivo.

### Análise estatística

Os eventos são expressos como números absolutos e porcentagens. As variáveis contínuas foram expressas em média ± desvio padrão (DP). Foi realizada uma análise descritiva dos dados. Os dados foram analisados utilizando o programa SPSS / PASW (IBM Corp, NY, USA).

## Resultados

Foram selecionados consecutivamente 51 pacientes (25 homens) para oclusão do AAE com o dispositivo LAmbre em 18 centros no Brasil. A idade média foi de 76 ± 7,7 anos. Os valores médios dos escores CHA2DS2-VASc e HAS-BLED foram 4,6±1,7 e 3,4±1,1, respectivamente. As indicações para o procedimento foram sangramento importante (principalmente cerebral ou gastrointestinal) em 24 pacientes (47,0%), AVC apesar de ACO oral adequada em 13 (25,5%) e sangramento e AVC em nove pacientes (17,6%). Outras indicações para oclusão do AAE foram contraindicação para ACO em três casos, trombo no AAE apesar de ACO adequada em um caso e escolha do paciente (por prática de esportes) em outro.

**Tabela 1:** – Características clínicas dos pacientes (n=51)

Variável	Resultado*
Idade (anos)	76 ± 7,7
Sexo feminino	26 (51)
Fibrilação atrial	
Permanente	26 (51)
Persistente	1 (2)
Paroxística	24 (47)
Escore CHA_2_DS_2_-VASc	4,6 ± 1,7
Escore HASBLED	3,4 ± 1,1
Indicações para oclusão do AAE	62 (68,1)
Sangramento importante	24 (47)
AVC apesar de TAO adequada	13 (25,5)
Sangramento + AVC	9 (17,6)
Contraindicação para TAO	3 (5,9)
Trombo persistente no AAE apesar de TAO adequada	1 (2)
Escolha do paciente	1 (2)

*Média ± DP ou números absolutos (porcentagem). AAE: apêndice atrial esquerdo; TAO: terapia de anticoagulação oral.

Os dados do procedimento estão descritos da Tabela 2 . O tamanho da zona de implante ( *landing zone* ) foi 23,84±4,5 mm e o tamanho médio do dispositivo implantado foi 27±5,1 mm – portanto, o tamanho do dispositivo implantado foi em média 3,7 mm maior do que a zona de implante do AAE. O tipo padrão do dispositivo o foi usado na maioria (94,1%) dos pacientes e o tipo especial usado nos demais (5,9%). Os tamanhos dos dispositivos padrão foi 28-34mm (n=9), 24-30mm (n=7), 30-34mm (n=6), 26-32mm (n=5), 34-38mm (n=5), 22-28mm (n=4), 32-36mm (n=4), 36-40mm (n=4), 18-24mm (n=2), e 20-26mm (n=2). Os tamanhos do dispositivo especial foram 16-30mm, 22-34mm e 24-36mm.

**Tabela 2:** – Dados dos procedimentos

Variável	Resultado*
Acesso	
Transeptal	48 (94,1)
FOP / DAS	3 (5,9)
Zona de implante (landing zone) (mm)	23,8 ± 4,5
Dispositivo implantado	
Tamanho (mm)	27 ± 5,1
Tipo padrão	48 (94,1)
Tipo especial	3 (5,9)
Dispositivos por procedimento (n)	
1	45 (88,2)
2	6 (11,8)
Sucesso	51 (100)
Shunt residual	
Nenhum	47 (92,2)
< 5mm	4 (7,8)
> 5mm	0
Complicações	
Óbito	0
AVC	0
Sangramento maior	0
Derrame pericárdico	2 (3,9)
Embolização (removido)	1 (2)

*Média ± DP ou números absolutos (porcentagem) FOP: forame oval patente; DAS: defeito do septo atrial; AVC: acidente vascular cerebral.

O primeiro dispositivo escolhido foi implantado em 45 pacientes (88,2%), e seis pacientes (11,8%) necessitaram de mais de um implante – em dois casos, o dispositivo primeiramente escolhido foi danificado durante o carregamento por operadores inexperientes e precisou ser substituído. Medidas incorretas levaram à necessidade de retirada e substituição do dispositivo por outro compatível com as dimensões do AAE em três pacientes. Um paciente apresentava AAE em formato de asa de galinha, e uma segunda punção transeptal em um ponto mais baixo, seguida de implante de um dispositivo menor, foi necessária para oclusão completa do apêndice.

**Figura 2:**
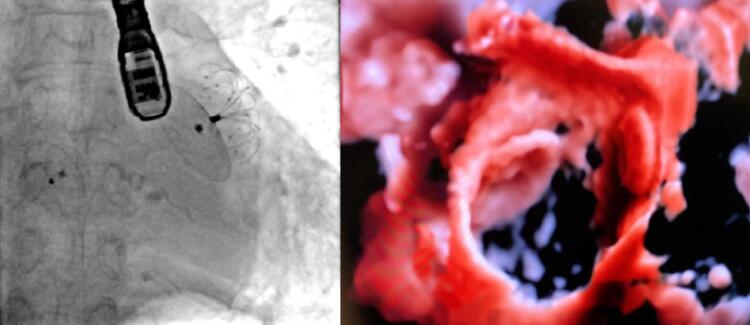
– Apêndice atrial esquerdo (AAE) fechado através do forame oval patente (FOP). À esquerda: dispositivo LAmbre ocluindo o AAE e um segundo dispositivo ocluindo o FOP. À direita: a mesma imagem vista por ecocardiografia esofágica 3D.

Três pacientes eram portadores de forame oval patente (FOP). Em dois desses pacientes, o acesso ao átrio esquerdo foi obtido através do FOP. O terceiro paciente apresentava o AAE em formato de asa de galinha, e impediu a coaxialidade da bainha de entrega. Realizou-se punção transeptal, e o procedimento foi conduzido sem maiores dificuldades. O FOP foi fechado em dois desses casos com dispositivos específicos (CERA PFO 25-18 mm em um caso; e CERA MF ASD 25-25mm no outro) ( Figura 2 ). Outro paciente apresentava um defeito do septo atrial, que foi ocluído durante o mesmo procedimento com um dispositivo Occlutech ASD 33mm.

Dois pacientes tiveram como complicação a formação de derrames pericárdicos. Em um deles, o apêndice foi perfurado pelo fio guia rígido. Realizou-se drenagem pericárdica imediata seguida pelo implante de uma prótese LAmbre 20-26mm que, no entanto, sofreu embolização imediata. Uma segunda prótese 34-38mm foi implantada com parada da progressão do derrame. A primeira prótese foi removida da aorta descendente no dia seguinte. Em um segundo paciente, derrame pericárdico com tamponamento cardíaco ocorreu poucas horas após o procedimento, devido à perfuração da artéria pulmonar principal pelos ganchos do dispositivo. Realizou-se drenagem cirúrgica, e o paciente se recuperou sem intercorrências.

**Figura 3:**
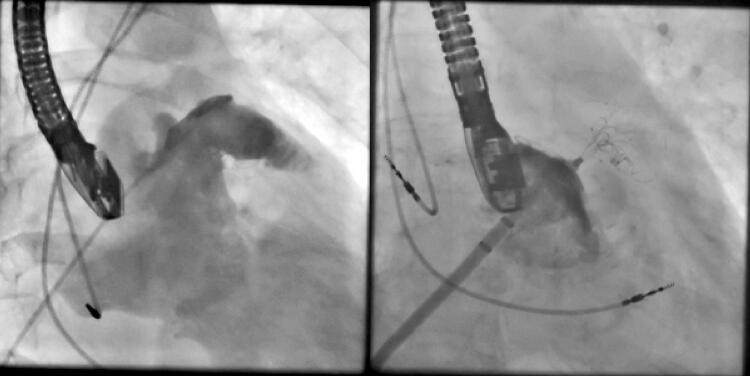
– À esquerda: Dispositivo Watchman implantado no lobo inferior do apêndice atrial esquerdo (AAE); o lobo superior permaneceu descoberto. À direita: dispositivo Watchman dentro do lobo inferior e o dispositivo LAmbre implantado no lobo direito com o disco ocluindo completamente o óstio do AAE. Não foi observado shunt residual imediatamente após o procedimento.

Um paciente foi submetido à oclusão do AAE com dispositivo Watchman, mas sofreu um AVC poucos meses depois devido à presença de um segundo grande lobo que inadvertidamente não foi abordado no primeiro procedimento. Meses depois, um dispositivo LAmbre foi implantado durante um segundo procedimento, com completa oclusão do AAE ( Figura 3 ).

**Figura 4:**
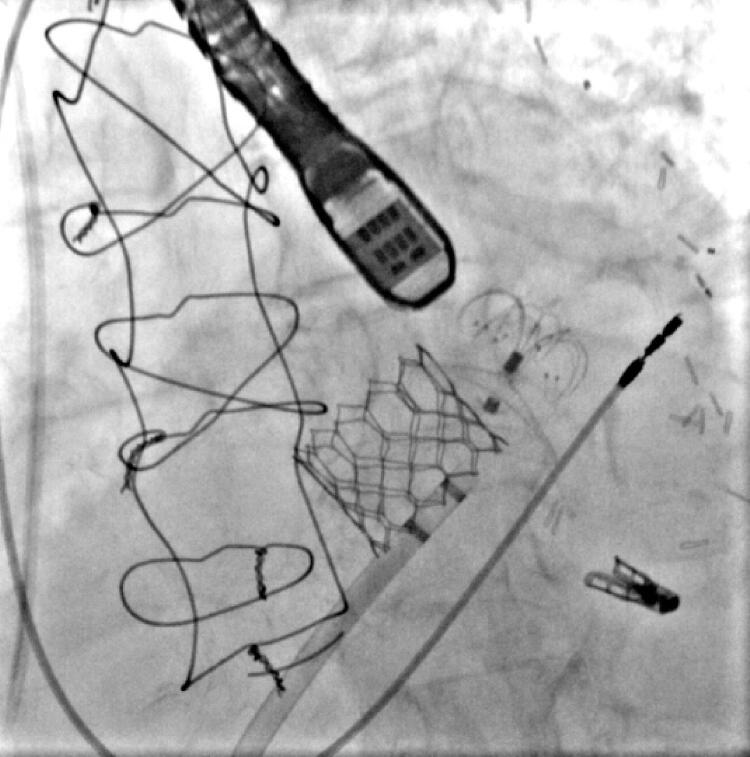
– Paciente de 86 anos de idade com várias intervenções: bypass coronário (CABG), implante de marcapasso, implante transcateter de válvula aórtica, Mitraclip e dispositivo LAmbre ocluindo o apêndice atrial. O implante do Mitraclip e a oclusão do apêndice atrial esquerdo foram realizados durante a mesma cirurgia.

Um paciente de 86 anos de idade havia se submetido à cirurgia de *bypass* coronário e implante de marcapasso. O paciente também apresentou estenose aórtica grave, que foi tratada com implante transcateter de válvula aórtica (TAVI). Uma semana após o TAVI, o paciente apresentou disfunção cardíaca grave devido à regurgitação mitral importante, e foi submetido ao implante de Mitraclip e LAmbre durante o mesmo procedimento cirúrgico ( Figura 4 ).

O implante do dispositivo foi possível em todos os casos. Não houve presença de shunts residuais significativos (>5 mm), embora shunts pequenos (<5mm) foram detectados por Doppler colorido em quatro pacientes (7,8%) ao final do procedimento. Nenhum paciente apresentou sangramento importante durante internação. Em um acompanhamento médio de 18 ± 12 meses, nenhum paciente sofreu outros episódios de sangramento importante ou tromboembolismo, e nenhum óbito ou complicações tardias foram relatados por nenhum centro.

## Discussão

**Figura 5:**
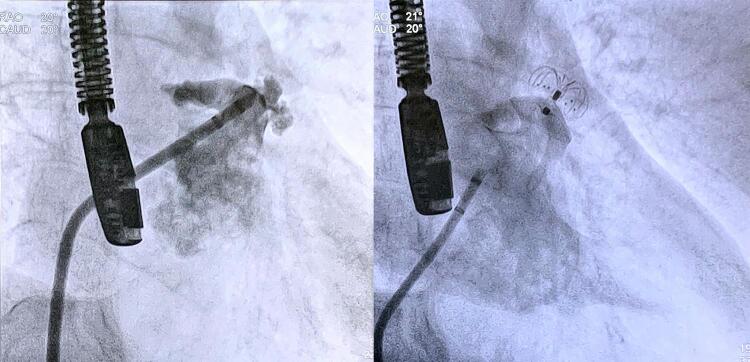
– À esquerda: apêndice atrial esquerdo (AAE) retrovertido, em formato de asa de galinha; à direita: AAE totalmente ocluído após implantação do dispositivo LAmbre (tipo padrão).

Inicialmente descrito por Lam em 2013,^12^ a prótese LAmbre foi descrita como um dispositivo de fácil utilização, seguro e eficaz. Vantagens potenciais do LAMbre em comparação a outros dispositivos foram destacadas pelo autor e incluíam a utilização de bainhas de entrega menores, a capacidade de ser totalmente retirado e reposicionado muitas vezes, e a maior estabilidade após seu implante. Ainda a possibilidade do implante proximal no AAE com pequeno número de manobras para reposicionamento, ajuda a prevenir a perfuração do AAE e possibilita o uso do dispositivo para tratamento dos AAEs com trombo distal por técnica “no-touch”, na qual o oclusor é implantado sem avançar a bainha de entrega ou o fio-guia no apêndice.^12 - 15^ O design dos dispositivos LAmbre (tanto o padrão como o especial) torna-o mais adequado em casos anatômicos particulares, principalmente na existência de zonas de implante ( *landing zones* ) rasas ou um desajuste entre um grande óstio e uma zona de implante estreita^16 , 17^ ( Figuras 5 e 6 ).

**Figura 6:**
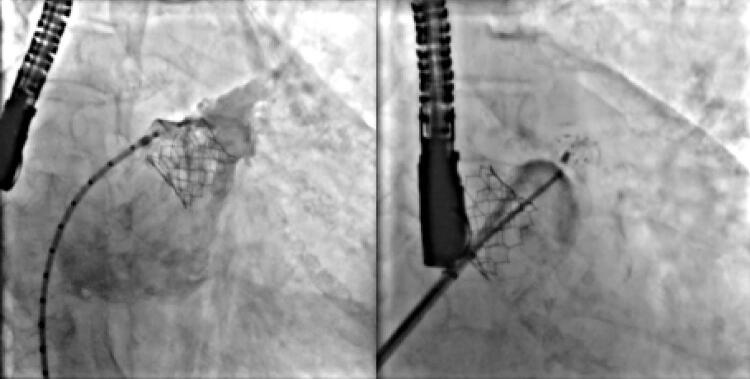
– À esquerda: apêndice atrial esquerdo (AAE) muito raso; à direita: AAE ocluído por um dispositivo LAmbre (tipo especial); esse paciente havia sido submetido à intervenção coronária percutânea e implante transcateter de válvula aórtica.

Apesar de mais de 7 000 implantes já terem sido realizado em todo o mundo, dados sobre o LAmbre® são ainda escassos na literatura. A publicação com o maior número de pacientes (n=153) mostrou uma taxa de complicações do procedimento de 3,3%, sem nenhum caso de embolização do dispositivo, e uma taxa anual de AVC de apenas 1,3% (vs. 6,4% predito pelo escore CHA_2_DS_2_-vasc) no seguimento.^11^ A experiência inicial europeia com 60 casos mostrou resultados similares (taxa de complicações de 3,3% e taxa anual de AVC de 1,6%).^18^

Uma revisão sistemática de 10 publicações, incluindo 403 pacientes com FANV tratados com LAmbre® apresentou uma taxa de sucesso do procedimento de 99,7% e uma taxa de complicações de 2,9% (0,3% de mortalidade, 1,7% de temponamento pericárdico, 0,3% AVC e complicações de sangramentos maiores), sem embolização do dispositivo. No seguimento, eventos adversos cardiovasculares maiores foram relatados em 3,3%; AVC ou ataque isquêmico transitório em 1,7%, formação de trombos no dispositivo em 0,7%, e fluxo residual > 5 mm em 1%.^19^

Um ensaio em andamento (Lifetech LAmbre^TM^Left Atrial Appendage Closure System Post-Market Clinical Follow-Up – LISA Study - NCT03122028) tem como objetivo recrutar 500 pacientes em 22 locais de oito diferentes países europeus e a China, com o propósito de avaliar a segurança e a viabilidade de implantes do dispositivo LAmbre em pacientes com FANV que não podem receber ACO. A comparação entre o LAmbre e o Amplatzer mostrou eficácia em longo prazo e segurança similares em pacientes com FANV.^20 , 21^

Os resultados imediatos e tardios do presente estudo estão de acordo com a literatura disponível. A taxa aceitável das complicações do procedimento e o seguimento favorável desta coorte de pacientes (de alto risco e com condição complexa) é encorajadora. As características únicas do dispositivo LAmbre®, principalmente de sua configuração especial, tornou factível e segura a realização de procedimentos desafiadores. A prótese LAmbre® traz avanços tanto no design do dispositivo como na técnica de implantação, e pode representar uma alternativa útil no armamentário de oclusão do AAE.

### Limitações

Este estudo tem várias limitações. Como uma limitação inerente a um estudo não randomizado, não há um grupo controle e, como em todo estudo observacional, falhas na seleção dos pacientes podem ter ocorrido. No entanto, este registro foi delineado para incluir todos os pacientes elegíveis para o procedimento (intenção de tratar), refletindo uma prática da vida real. Embora os dados tenham sido coletados prospectivamente, esta é uma análise retrospectiva, sem monitoramento independente ou análise de um laboratório central. Devido a dificuldades de reembolso no Brasil, basicamente todos os centros incluídos neste registro têm um baixo volume de oclusão de AAE e, assim, a curva de aprendizagem dos operadores é achatada, o que tem um impacto direto sobre as taxas de complicação. Por fim, todos os dados coletados foram espontaneamente relatados pelos investigadores, sem uma avaliação independente.

## Conclusões

A experiência inicial com o dispositivo LAmbre em 18 centros brasileiros foi segura e eficaz, neste pequeno número de pacientes. Assim como em todos os dispositivos usados para a oclusão do AAE, a curva de aprendizado com o LAmbre teve um impacto sobre as complicações, e taxas aceitáveis e comparáveis às da literatura. No entanto, são necessários um maior número de pacientes e um seguimento mais longo para se obter uma comparação mais apropriada entre o LAmbre e outros dispositivos atualmente utilizados para oclusão do AAE no Brasil.
