# Supplementary material for: Oclusão do Apêndice Atrial Esquerdo com a Prótese Lambre: Experiência Multicêntrica Inicial no Brasil
Source: Arq Bras Cardiol. 2022 Apr 26;119(1):48–56. [Article in Portuguese] doi: 10.36660/abc.20210275 (PMC9352115; doi:10.36660/abc.20210275)
Supplement: Translation

# Translation: Left Atrial Appendage Closure with the LAmbre Device – Initial Multicentre Experience in Brazil

**Authors:** Francisco Chamié, Enio Guerios, Daniel Peralta e Silva, Valério Fuks, Rômulo Torres

**Affiliations:** 1 Brazil Hospital Federal dos Servidores do Estado – Cardiologia Intervencionista, Rio de Janeiro, RJ – Brazil; 2 Brazil INTERCAT - Cardiologia Intervencionista, Rio de Janeiro, RJ – Brazil; 3 Brazil Universidade Federal do Paraná – Hospital de Clínicas, Curitiba, PR – Brazil; 4 Brazil Hospital Marcelino Champagnat, Curitiba, PR – Brazil

**Keywords:** Atrial Fibrillation, Atrial Appendage, Coronary Occlusion

## Abstract

**Background:**

Left atrial appendage (LAA) closure has been an alternative to oral anticoagulation (OAC) for stroke prevention in patients with non-valvular atrial fibrillation (NVAF).

**Objectives:**

To report the first results of an initial multicenter experience in Brazil and to investigate the feasibility, safety, and efficacy of LAA closure with the new LAmbre device.

**Methods:**

We collected procedural and follow-up data of 51 consecutive patients with non-valvular atrial fibrillation, restrictions for long-term OAC and suitable anatomy that underwent LAA closure with the LAmbre device in 18 centers in Brazil. Procedural indications were significant bleeding under OAC (47.1%), stroke or persistent LAA thrombus despite OAC (27.5%), bleeding plus stroke (17.6%), other clinical contraindications for OAC (5.9%), and patient’s choice due to sports practice (1.9%).

**Results:**

Twenty-five men (49%) and 26 women (51%), with a mean age of 76±7.7 years, mean CHA2DS2-VASc score of 4.6± 1.7 and mean HAS-BLED score of 3.4± 1.1 were studied. Procedural success rate was 100%. Procedure-related immediate complications were pericardial effusion in two patients, and immediate device embolization in one case. No large residual shunts (> 5 mm) were observed, and small shunts (<5mm) were detected in four patients by color Doppler at the end of the procedure. After a mean follow-up of 18 ± 12 months, there were no deaths, strokes nor any other major complications.

**Conclusion:**

LAA occlusion with the LAmbre device was safe and effective in this small case series. Despite these encouraging initial results, the small number of cases warrants further studies with longer-term follow-up.

## Introduction

Transcatheter left atrial appendage (LAA) occlusion has become increasingly popular as an alternative option to anticoagulation for thromboembolic event prophylaxis in non-valvular atrial fibrillation (NVAF) patients.^1^Oral anticoagulant therapy (OAC), either with Vitamin K Antagonists (VKA) or Direct Oral Anticoagulants (DOACs) has proven to be an effective therapy for preventing stroke in AF patients.^2^Unfortunately, long-term OAC compliance, underprescription, and complications make this therapy not applicable for a significant number of patients.^3^Thus, the need for a non-pharmacological form of prophylaxis has increased over the years.

Considering that over 90% of the intra-atrial thrombi formed as a consequence of NVAF are located inside the trabecular portion of the atrial appendage, LAA occlusion seemed to be a reasonable option.^4^Initially proposed as a surgical procedure, percutaneous occlusion is now performed all over the world. Different devices and techniques are available for LAA occlusion. Randomized trials have shown that LAA occlusion is non-inferior to Warfarin and DOACs in terms of reduction of stroke and systemic embolism, and superior to warfarin regarding late mortality.^5 - 10^

LAmbre device is a LAA occluder released in Brazil in 2018, after the Amplatzer Cardiac Plug (ACP) and the Watchman Filter devices. This paper intends to report the results of the first multicenter Brazilian Registry of the use of the LAmbre device for percutaneous closure of the LAA for stroke prevention in patients with NVAF.

## Methods

Between May 2018 and November 2020 consecutive patients who underwent percutaneous LAA closure with the LAmbre device in 18 different centers in Brazil were prospectively studied. Many of these procedures were performed under the supervision of a proctor. All patients had NVAF and an absolute or relative contraindication for long-term OAC therapy – the only exception was one patient who refused OAC due to personal preferences. All of them had been submitted to transesophageal echocardiogram (TEE) or cardiac computed tomography (CT) for assessment of LAA size and morphology, intended landing zone diameters and the presence of thrombus.

The procedures were performed under general anesthesia and orotracheal intubation. Non-fractioned heparin (100 mg/kg or 10,000 International Units) and antibiotic prophylaxis (intravenous Cefazolin 2g) were administered to all patients, followed by Cefazolin 1g IV as single dose 6 h after procedure, in the intensive care unit (ICU). Procedures were monitored by TEE and fluoroscopy.

After femoral venous access was obtained, transseptal puncture with Brockenbrough needle was done targeting the inferior and posterior fossa ovalis. Left atrial (LA) pressure was recorded immediately after the left atrium was accessed; if values were lower than 10 mmHg, saline was rapidly infused for restoration of true LAA diameters. A 5F Pigtail catheter was positioned inside the LAA to obtain angiographies and measurements in right anterior oblique (RAO) caudal and cranial views. After angiography, a Super-Stiff guidewire J-Tip 0.035”/260 cm was cautiously introduced inside the LAA through the Pigtail catheter. The device size was confirmed by intraoperative angiography and TEE and should be 2 to 8 mm larger than the intended landing zone. The device was implanted through the long sheath indicated for the size of the chosen device. Implant technique was described elsewhere.^11^

Patients were kept in ICU overnight and discharged from hospital the next day after another TTE, provided no complications occurred.

Aspirin (100 mg) and Clopidogrel (75 mg) were prescribed after procedure. Clopidogrel was discontinued after three months, and lifelong Aspirin prescribed thereafter. Follow-up TEE was performed three and six-months after the procedure.

### The Lambre Device

The LAmbre^TM^device (Lifetech Scientific, Shenzhen, China) is a self-expanding Titanium Nitride (TiN)-covered nitinol mesh occlusion device. It comprises three parts: a disc designed to cover the LAA ostium, a connector pin, and an eight-armed umbrella with small attachment hooks, that anchors the device to the body of the LAA, increasing stability. The umbrella is designed with a forward movement of the arms whose atraumatic tips, when fully opened, engage the trabeculae of the LAA, and the small distal hooks connect to the LAA wall, enhancing the stability of the device. The disk is configured to totally cover the LAA ostium. Both umbrella and disk have polyethylene terephthalate fabric sewn inside ( Figure 1 ). In addition, LAmbre^TM^device comes in two versions: the standard type and the special type device.

**Figure 1:** – LAmbreTM device. Left panel shows umbrella and disk connected through a central pin. The arms have atraumatic round tips that engage the trabecular portion of the left atrial appendage (LAA) and small hooks that attach to the LAA wall. The disk covers the LAA ostium and is connected to the umbrella by a pin, with no screw protruding on the external surface of the disk. Right panel: fluoroscopy after implantation.

In the standard type device, the umbrella sizes range from 16 to 36 mm in two-millimeter increments, with disks that are 6 mm larger than 16-30mm umbrellas or 4 mm larger than 32-36mm umbrellas. In the special type device, the umbrella sizes range from 16 to 26 mm, also in two-millimeter increments, with disks 14 mm larger than 16-18 mm and 12 mm larger than 20-26 mm umbrellas.

The delivery system is composed of a double curve (45 and 30 degrees), sheath of 8F to 10F, and a delivery cable with screwing mechanism. Its noteworthy that the screw on the disk surface is recessed, to prevent thrombus formation over the device.

### Statistical analysis

Events are expressed as absolute numbers and percentages. Continuous variables were expressed as mean ± standard deviation (SD). A descriptive analysis of the data was carried out. Data were analyzed using the software SPSS / PASW (IBM Corp, NY, USA).

## Results

A total of 51 patients (25 men) were consecutively selected for LAA occlusion with the LAmbre device in 18 different centers in Brazil. Mean age was 76±7.7 years. Mean CHA2DS2-VASc and HAS-BLED were 4.6±1.7 and 3.4±1.1, respectively. AF was paroxistic in 24, persistent in 1 and permanent in 26 patients. Indications for the procedure were significant bleeding (mostly cerebral or gastrointestinal) in 24 patients (47.0%), stroke despite adequate OAC in 13 (25.5%) and bleeding and stroke in nine (17.6%) patients. Other indications for LAA occlusion were contraindication to OAC in three cases, persistent LAA thrombus despite OAC in one case and patient’s choice (due to sports practice) in another ( Table 1 ).

**Table 1:** – Clinical characteristics of the patients (n=51)

Variable	Result*
Age (years)	76 ± 7.7
Female sex	26 (51)
Atrial fibrillation	
Permanent	26 (51)
Persistent	1 (2)
Paroxystic	24 (47)
CHA_2_DS_2_-VASc Score	4.6 ± 1.7
HASBLED Score	3.4 ± 1.1
Indications for LAA closure	62 (68,1)
Significant bleeding	24 (47)
Stroke despite adequate OAC	13 (25.5)
Bleeding + stroke	9 (17.6)
Contraindication for OAC	3 (5.9)
Persistent thrombus in LAA despite OAC	1 (2)
Patient’s choice	1 (2)

*Mean ± SD or absolute numbers (percentage). LAA: left atrial appendage; OAC: oral anticoagulation.

Procedural data are presented in Table 2 . Mean landing zone size was 23.84±4.5 mm and mean size of the implanted device was 27±5.1 mm – thus the size of the implant was 3.7mm (mean) larger than the measured LAA orifice. Standard type device was used in the majority (94.1%) of patients and the special type in the remainder (5.9%). The sizes of the implanted devices were 28-34mm (n=9), 24-30mm (n=7), 30-34mm (n=6), 26-32mm (n=5), 34-38mm (n=5), 22-28mm (n=4), 32-36mm (n=4), 36-40mm (n=4), 18-24mm (n=2), e 20-26mm (n=2). Special device sizes used were 16-30mm, 22-34mm and 24-36mm.

**Table 2:** – Procedural data

Variable	Result*
Access	
Transeptal	48 (94.1)
PFO / ASD	3 (5.9)
Landing zone (mm)	23.8 ± 4.5
Device implanted	
Size (mm)	27 ± 5.1
Standard design	48 (94.1)
Special design	3 (5.9)
Devices per procedure (n)	
1	45 (88.2)
2	6 (11.8)
Success	51 (100)
Residual leak	
None	47 (92.2)
Minor (< 5mm)	4 (7.8)
Major (> 5mm)	0
Complications	
Death	0
Stroke	0
Major bleeding	0
Pericardial effusion	2 (3.9)
Embolization (snared)	1 (2)

*Mean ± SD or absolute numbers (percentage). PFO: patent foramen ovale; ASD: atrial septal defect.

The first chosen device was implanted in 45 patients (88.2%). A second device was necessary in six patients (11.8%): In two cases the first chosen device was damaged during loading by inexperienced operators and needed to be replaced. Incorrect measurements determined device retrieval and replacement for another one more compatible with LAA dimensions in three patients. In another case with challenging anatomy due to a retroflexed chicken wing appendage, a second lower transeptal puncture and smaller device implantation was deemed necessary to achieve total occlusion.

In addition, three patients had patent foramen ovale (PFO). In two of them, access to left atrium was obtained through the PFO tunnel. The third one had a retroflex chicken wing appendage and access via PFO tunnel prevented delivery sheath coaxiality. Transseptal puncture was performed, and the procedure was carried out without further difficulty. The PFO was closed in two of these cases with a dedicated device (25-18 mm CERA PFO device in one case and 25-25 mm CERA MF ASD device in the other) ( Figure 2 ). Another patient had an ostium secundum atrial septal defect that was closed in the same procedure with a 33mm Occlutech ASD device.

**Figure 2:** – Left atrial appendage (LAAE) closed through a patent foramen ovale (PFO). Left: LAmbre device occluding the LAA and a second dedicated device occluding the PFO; right: the same image seen by 3D transesophageal echocardiogram .

Two patients developed pericardial effusions. In one of them the appendage was perforated by the stiff guidewire. Percutaneous pericardial drainage was immediately carried out and was followed by a LAmbre 20-26mm device implantation, which was immediately embolized. A second 34-38mm device was implanted and the effusion subsided. The first device was snared out from the descending aorta the next day. In a second patient pericardial effusion with cardiac tamponade occurred few hours after the procedure, due to perforation of the main pulmonary artery by the hooks of the device. Surgical drainage was performed, and the patient recovered uneventfully.

One patient underwent previous LAA closure with a Watchman device, but suffered a recurrent stroke few months thereafter due to a second large lobe that was inadvertently left uncovered in the first procedure. A LAmbre device was implanted in a second procedure months later with total occlusion of LAA ( Figure 3 ).

**Figure 3:** – Left: Watchman device implanted in a lower lobe of the left atrial appendage (LAA); the upper lobe was left uncovered. Right: Watchman device inside the lower lobe and the LAmbre device implanted in the upper lobe with the disk totally occluding the ostium of the LAA. No residual shunt was seen immediately after the procedure.

An 86-year-old male patient had been previously submitted to coronary bypass graft surgery with pacemaker implantation. He also had severe aortic stenosis, which was treated with transcatheter aortic valve implantation (TAVI). One week after TAVI, the patient presented with important cardiac dysfunction due to significant mitral regurgitation and had Mitraclip and LAmbre implanted during the same surgical procedure ( Figure 4 ).

**Figure 4:** – Eighty-six-year-old patient with multiple interventions: coronary artery bypass graft, pacemaker implantation, transcatheter aortic valve implantation (TAVI), Mitraclip and LAmbre device occluding the atrial appendage. Both Mitraclip implantation and the left atrial appendage occlusion were performed during the same surgical procedure.

Device implantation was possible in all cases. There were no large residual shunts (> 5 mm) and minor shunts (<5mm) were detected by color Doppler in four patients (7.8%) at the end of the procedure. No patient had significant bleeding during hospitalization. During a mean follow-up of 18±12 months, none of the patients suffered further significant bleeding or thromboembolic events, and no deaths or late complications were reported by any center.

## Discussion

Initially described by Lam in 2013,^12^the LAmbre was described as an easy-to-use, safe and effective device. Potential advantages of LAmbre over other devices were pointed out by the author and included smaller delivery sheaths, the ability to be fully retrieved and repositioned many times and enhanced stability after implantation. Moreover, the possibility of shallow device deployment and the use of less maneuvers for positioning helps to prevent LAA perforation and enables the use of the device for treatment of LAAs with distal thrombus using the no-touch technique, in which the occluder is implanted without advancing neither the delivery sheath nor the guidewire into the appendage.^12 - 15^The design of the standard and special LAmbre devices makes it more suitable in case of difficult anatomies, mainly when there are shallow landing zones or a mismatch between a large ostium and narrow landing zone^16 , 17^( Figures 5 and 6 ).

**Figure 5:** – Left: retroflexed chicken wing left atrial appendage (LAA); right: LAA totally occluded after implantation of a standard-type LAmbre device.

**Figure 6:** – Left: very shallow left atrial appendage (LAA); right: LAA occluded by a special-type LAmbre device. Additionally, this patient was previously submitted to percutaneous coronary intervention and *transcatheter aortic valve implantation.*

Although more than 7,000 implants have already been made worldwide, literature on LAmbre^TM^is still scarce. The publication with the largest number of patients (n=153) showed a 3.3% procedural complication rate, with no case of device embolization, and a yearly stroke rate of only 1.3% (vs. 6.4% predicted by the CHA_2_DS_2_-vasc score) at follow up.^11^The initial European experience with 60 cases had similar results (procedural complication rate of 3.3%, annual stroke rate at follow-up of 1.6%).^18^

A systematic review of 10 publications encompassing 403 NVAF patients treated with LAmbre showed a procedural success rate of 99.7% and an overall complication rate of 2.9% (0.3% mortality, 1.7% pericardial tamponade, 0.3% stroke and major bleeding complications) with no device embolization. At follow-up, major adverse cardiovascular events were reported in 3.3%; stroke or transient ischemic attack in 1.7%, thrombus formation on the device in 0.7% and residual flow > 5mm in 1%.^19^

An ongoing trial (Lifetech LAmbre^TM^Left Atrial Appendage Closure System Post-Market Clinical Follow-Up – LISA Study; NCT03122028) aims to enroll 500 patients in 22 study sites in eight different countries in Europe and China, with the purpose to examine the safety and feasibility of LAmbre device implantations in patients with NVAF that cannot use OAC. Comparison between LAmbre and Amplatzer devices showed similar long-term efficacy and safety in patients with NVAF.^20 , 21^

The immediate and late results presented in this study are well in accordance with available literature. The acceptable rate of procedural complications and the favorable follow-up of this high-risk and complex cohort of patients is encouraging. The unique features of the LAmbre^TM^device, most particularly in its special configuration, rendered very challenging procedures safely feasible. This prosthesis brings advances in both device design and implantation technique and may be a valuable addition to the armamentarium of LAA closure.

### Limitations

This study has several limitations. As an inherent limitation to a non-randomized study, there is no control group. As in every observational study, there may be flaws in patient selection. However, this registry was designed to include all patients eligible for the procedure (intention-to-treat), reflecting a real-world practice. Although the data have been prospectively collected, this is a retrospective analysis, without independent monitoring, or a core lab analysis. Especially due to reimbursement difficulties in Brazil, basically all centers included in this Registry are centers with low volume of LAA closure and, thus, the learning curve of the operators is flattened, which has a direct impact on complication rates. And, finally, all the data collected were spontaneously reported by investigators, without independent adjudication.

## Conclusions

Initial experience with the LAmbre device in 18 different centers in Brazil was safe and effective, in this small number of patients. As with all devices used for LAA closure, the learning curve with LAmbre had an impact on complications, but even so at rates that are acceptable and comparable to the literature. Be it as it may, a larger number of patients and a longer- term follow-up is warranted for obtaining a fair comparison between LAmbre and the other devices currently used for percutaneous LAA closure in Brazil.
